# Characteristics of nursing homes with high rates of invasive methicillin‐resistant *Staphylococcus aureus* infections

**DOI:** 10.1111/jgs.19189

**Published:** 2025-01-20

**Authors:** Isaac See, Kelly A. Jackson, Kelly M. Hatfield, Prabasaj Paul, Rongxia Li, Joelle Nadle, Susan Petit, Susan M. Ray, Lee H. Harrison, Laura Jeffrey, Ruth Lynfield, Carmen Bernu, Ghinwa Dumyati, Anita Gellert, William Schaffner, Tiffanie Markus, Runa H. Gokhale, Nimalie D. Stone, Kara Jacobs Slifka

**Affiliations:** ^1^ Division of Healthcare Quality Promotion Centers for Disease Control and Prevention Atlanta Georgia USA; ^2^ Healthcare‐associated Infections California Emerging Infections Program Oakland California USA; ^3^ Infectious Diseases Section Connecticut Department of Public Health Hartford Connecticut USA; ^4^ Division of Infectious Diseases Emory University School of Medicine Atlanta Georgia USA; ^5^ Department of International Health Johns Hopkins Bloomberg School of Public Health Baltimore Maryland USA; ^6^ Division of Infectious Diseases University of Pittsburgh School of Medicine Pittsburgh Pennsylvania USA; ^7^ Emerging Infections Program Minnesota Department of Health St. Paul Minnesota USA; ^8^ Center for Community Health and Prevention University of Rochester Medical Center Rochester New York USA; ^9^ Department of Health Policy Vanderbilt University Medical Center Nashville Tennessee USA

**Keywords:** infection control, methicillin‐resistant *Staphylococcus aureus*, nursing homes, public health

## Abstract

**Background:**

Nursing home residents experience a large burden of invasive methicillin‐resistant *Staphylococcus aureus* (MRSA) infections. Data are limited regarding nursing home characteristics associated with differences in facility‐level invasive MRSA rates.

**Methods:**

We analyzed 2011–2015 data from CDC's Emerging Infections Program (EIP) active population‐ and laboratory‐based surveillance for invasive MRSA cases within seven states. A nursing home‐onset case was defined as MRSA cultured from a normally sterile site in a person living in a nursing home 3 days before culture collection. Facility rates were calculated as nursing home‐onset cases per 100,000 resident‐days. Nursing home resident‐day denominators and facility characteristics were obtained from four Centers for Medicare & Medicaid Services (CMS) datasets. A general estimating equations model with a logit link assessed characteristics of the facilities with highest rates comprising 50% of nursing home MRSA cases (“high rates”).

**Results:**

The 626 nursing homes in the surveillance area had 2824 invasive MRSA cases; 82% of facilities had at ≥1 case. The 20% of facilities with highest rates (≥3.84 cases/100,000 resident‐days) had 50% of nursing home‐onset cases. In multivariable regression, facilities with high rates were more likely to have CMS‐derived characteristics of presence of a resident with a multidrug‐resistant organism; or greater proportions of residents who were male, were short stay (in the facility <100 days), had a nasogastric or percutaneous gastrostomy tube, or require extensive assistance with bed repositioning; and more likely to be in an EIP area with higher hospital‐onset MRSA rates. Higher registered nurses staffing levels (hours/resident/day) and higher proportions of White residents were associated with lower rates.

**Conclusions:**

Facilities with higher invasive MRSA rates served residents with more clinical and functional care needs. Increasing registered nurse staffing in high‐risk facilities might assist with reduction of invasive MRSA rates. These findings could help prioritize nursing homes for future MRSA prevention work.

**Key points**

- 82% of nursing homes included in the project during 2011–2015 had at least one resident with an invasive MRSA infection.
- The 20% of nursing homes with the highest invasive MRSA rates accounted for 50% of invasive MRSA infections in nursing homes.
- Nursing homes with higher invasive MRSA rates served residents with more clinical and functional care needs and had lower levels of registered nurse staffing.

**Why does this paper matter?**

These findings can help focus infection prevention actions in nursing homes caring for residents with high likelihood of developing invasive MRSA.

## INTRODUCTION

Methicillin‐resistant *Staphylococcus aureus* (MRSA) has long been recognized as a serious pathogen affecting patients in hospitals.^1^ Following initial recognition of MRSA as an important source of community‐acquired infections,^2^, ^3^ surveillance data indicate that incidence of hospital‐onset MRSA bloodstream infections has declined by 74% in the United States.^4^

Health care in the United States has also evolved. The average length of a hospital admission shortened during the 1990s and 2000s,^5^, ^6^ and the proportion of hospitalizations resulting in discharge to post‐acute‐care facilities such as skilled nursing facilities for continued care has increased over time.^7^ Several studies have identified a high prevalence of MRSA colonization in nursing home residents. A review reported that 8%–10% of all US nursing home residents are colonized with MRSA.^8^ However, some studies have found colonization prevalence among residents exceeding 40% in certain facilities.^9^, ^10^

In accordance with these shifts in MRSA epidemiology and in healthcare utilization, in regions where the CDC's Emerging Infections Program (EIP) conducts surveillance for MRSA, a higher burden of nursing home‐onset invasive MRSA infections compared with hospital‐onset infections has been observed.^11^ That analysis found that of nursing home‐onset invasive MRSA infections, the most common infection syndromes were bloodstream infection (91%, including 41% bloodstream infection alone), pneumonia (21%), urinary tract infection (9%), bone or joint infection (8%), and pressure ulcer or chronic wound infection (7%). It also found that aggregated rates of invasive MRSA infections in nursing homes varied across surveillance sites, but data were not available to determine how such rates varied across facilities. Understanding the reasons for these variations could help identify facilities that would most benefit from additional MRSA prevention efforts.

We investigated the variation in nursing home‐onset invasive MRSA rates at the facility level and sought to describe which facility‐level characteristics were associated with high MRSA rates.

## METHODS

### EIP data

Data on invasive MRSA infections were obtained from CDC's EIP surveillance in selected counties in seven sites reporting data from 2011 to 2015 (California, Connecticut, Minnesota, New York, Tennessee), 2012–2015 (Georgia), and 2011–2014 (Maryland). Details about the surveillance can be found elsewhere.^11^ Briefly, this is an active laboratory‐ and population‐based (i.e., surveillance for cases in a specified geographic area) public health surveillance program; a case is defined as identification of MRSA from a normally sterile body site in a resident of the surveillance area. Epidemiologic data are collected through medical record review.

For cases occurring in 2011–2015, EIP site staff routinely determined during medical record review whether cases resided in a “long‐term care facility” 3 days before the initial invasive MRSA culture was obtained. A long‐term care facility was defined as a freestanding nursing home, an inpatient rehabilitation facility, or an inpatient hospice facility.

For this project, EIP site staff retrospectively determined whether the long‐term care facility was a nursing home, and if it was, documented the specific nursing home the patient resided in. A complete list of nursing homes certified by the Centers for Medicare & Medicaid Services (CMS) within the surveillance area was extracted from the CMS Provider of Services files from 2011 to 2015^12^ (Appendix). Sites were provided a list of nursing homes and addresses from the CMS Provider of Services file to guide the retrospective review. Nursing home‐onset cases were defined as cases in which the patient was documented to be a resident of a nursing home 3 days before the collection date of the initial invasive MRSA culture.

### Facility characteristic data

Resident‐day denominator data for facilities within the EIP MRSA surveillance area were obtained from CMS skilled nursing facility cost reports as described previously.^11^, ^13^, ^14^

Two other data sources were used to obtain information about facility characteristics. Information about nursing home residents was obtained from the CMS Minimum Data Set (MDS).^15^ For each facility, variables were created summarizing the percentage of residents with specified characteristics based on the most recent MDS assessment available for the residents as of April 2, 2015. A single date was chosen to characterize the facility because of patterns in the data suggesting changes in reporting behavior during the analytic period. In addition, the June 2015 CMS nursing home compare data were used to obtain information about nursing home staffing and ratings on CMS quality measures.^16^

A complete list of facility characteristics initially considered for analyses can be found in the Appendix. In summary, the characteristics were related to the following concepts: distribution of short‐stay patients, patient demographics, patient comorbidities (including presence of wounds or multidrug‐resistant organisms), patient's functional status, medical device usage, antibiotic use, staffing, and quality of care measures. To reduce the number of variables ultimately included in analyses, a large number of potential characteristics from the data sources listed above were categorized by internal review according to the mechanism by which they might influence invasive MRSA rates. If two different variables were considered to represent the same concept, the characteristic considered to have the most straightforward interpretation was selected to include in the analysis. Details of this process can also be found in the Appendix.

From the Minimum Data Set, the variables selected were the percentage of residents that were White, were African American, male, had a multidrug‐resistant organism, had a tracheostomy, had a nasogastric or percutaneous gastrostomy tube, had a wound, received antibiotics recently, were on dialysis, had diabetes, were short stay (i.e., in the facility less than 100 days), or needed extensive assistance for bed repositioning. For variables in which the 75th percentile of facilities had no residents with that characteristic, the variable was dichotomized as a binary variable indicating just presence or absence of at least one resident with that characteristic. Otherwise, variables from the Minimum Dataset are used in the model in their original continuous form. From CMS nursing home compare registered nurse staffing (hours/resident/day), CMS survey rating, and the CMS quality measure rating were included for analysis.^16^

In addition, the rate of hospital‐onset invasive MRSA (cases with MRSA cultured on hospital admission day 4 or later) for each of the seven EIP sites was calculated as the number of hospital‐onset cases divided by the census population. For each facility, the hospital‐onset rates for the overlying site for 2014 were included in the model as a covariate to assess the potential relationship between MRSA activity in surrounding hospitals and nursing home‐onset invasive MRSA rates. Community‐associated invasive MRSA (i.e., cases that are not hospital‐onset in patients without a major preceding healthcare exposure) rates were not included because of insufficient variation in rates across EIP sites (Supplemental Figure 1).

### Data analysis

Missing resident‐days for facilities were imputed using the predictive mean matching imputation method,^17^, ^18^ based on facility bed size.^11^ Additional details can be found in the Appendix.

Facility‐level nursing home‐onset invasive MRSA rates were calculated as the number of invasive MRSA nursing home‐onset cases present in that facility divided by the total number of resident‐days. Nursing homes within the surveillance area were included in the analysis if they were open during 2015 and reported data to the CMS MDS in 2015. Nursing homes were ranked from highest to lowest invasive MRSA rates. To identify facilities with high rates contributing the most to nursing home‐onset invasive MRSA burden, the facilities with highest rates that cumulatively had >50% of the invasive MRSA case burden were classified as having “high” invasive MRSA rates.

A descriptive analysis compared characteristics of facilities with high invasive MRSA rates with other facilities. To determine statistical significance at *p* < 0.05, a Wilcoxon rank‐sum test was used to compare median of continuous variables between higher and lower MRSA rate groups, such as % of White residents. A chi‐square test was used to test the association between categorical variables (e.g., the EIP site) and higher or lower rate groups. The distribution of characteristics (i.e., 25th percentile, median, 75th percentile) across facilities was also summarized for each continuous variable.

A generalized estimating equations model with a logit link and state as the level of clustering was used to calculate the adjusted odds ratio (aOR) and corresponding 95% confidence interval (CI) for the associations between facility characteristics and having a high invasive MRSA rate.

Analyses were performed using SAS, version 9.4 (SAS Institute, Inc., Cary, NC).

This activity was reviewed by CDC and was conducted consistent with applicable federal law and CDC policy.^1^ EIP sites obtained local human subjects approvals including institutional board review (IRB) if required.

## RESULTS

During the project period, 3074 invasive MRSA cases occurred among residents in long‐term care facilities 3 days prior to the initial invasive MRSA culture. For 250 cases, the medical record was unavailable to determine whether the long‐term care facility was a nursing home, the facility was determined not to be a nursing home in the surveillance area, or the facility was referred to as a nursing home in the medical record, but the specific facility was not identified. In total, 2824 were nursing home‐onset MRSA cases linked to a specific nursing home.

Of the 666 nursing homes located in the 7‐site surveillance area during the project period, 38 closed before 2015 and two did not have data in MDS. Consequently, data from 626 facilities were eligible for inclusion in the analysis.

**FIGURE 1:**
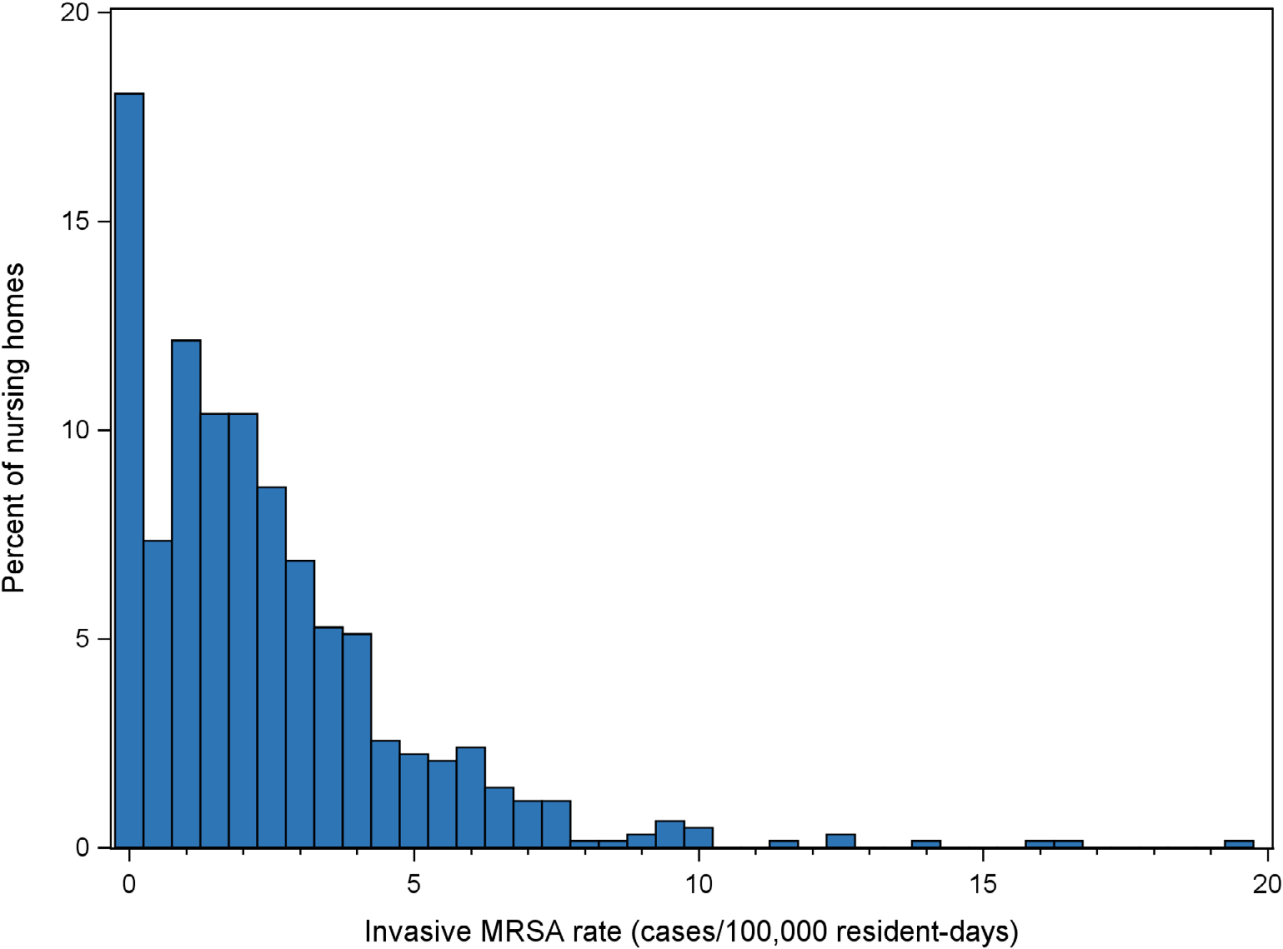
Distribution of nursing home‐onset invasive methicillin‐resistant *Staphylococcus aureus* (MRSA) rates, Emerging Infection Program surveillance area, 2011–2015.

**FIGURE 2:**
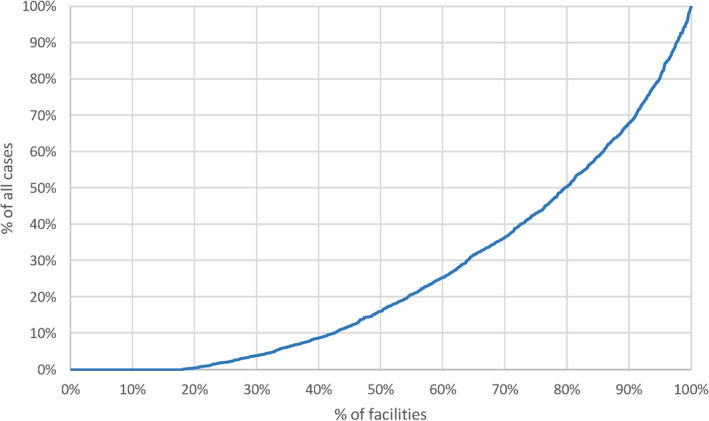
Cumulative burden of invasive nursing home‐onset methicillin‐resistant *Staphylococcus aureus* (MRSA) cases as facility MRSA rate increases, Emerging Infection Program surveillance area, 2011–2015. Approximately 50% of the total MRSA case burden occurs in the 20% of facilities with the highest rates.

Of these 626 nursing homes, 82% had at least one resident with an invasive MRSA infection during the project period (Figure 1). It was found that the 20% of facilities (*n* = 127) with the highest rates (i.e., those with “high rates”) accounted for 50% of the invasive MRSA cases. These facilities had rates of at least 3.84 cases/100,000 resident‐days (Figure 2). Among all 626 nursing homes, the median invasive MRSA rate was 1.82 cases/100,000 resident‐days, with a maximum of 19.3 and an interquartile range of 0.74–3.37 cases/100,000 resident‐days. Across these facilities, the median number of invasive MRSA cases per facility was 3 (range: 0–36 cases, interquartile range: 1–6 cases) (Supplemental Figure 2).

A univariate assessment of the characteristics of facilities with high rates vs other facilities is displayed in Table 1. There was a significant difference in geographic distribution (*p* < 0.0001 for the overall chi‐square test of site vs nursing home‐onset invasive MRSA rate). For example, only 12% of the facilities in the project were in Maryland, but 28% of the facilities with high invasive MRSA rates were in Maryland. All facility‐level characteristics were significantly associated with differences in rates in univariate analysis except for registered nurse (RN) staffing (*p* = 0.74) and CMS quality measure rating (*p* = 0.47). The minimum, median, quartile range, and maximum value of facility characteristics are shown in Table 2.

**TABLE 1:** Descriptive characteristics of nursing homes with higher vs lower rates of invasive methicillin‐resistant *Staphylococcus aureus* infections, Emerging Infections Program (EIP) surveillance area, 2011–2015.

Characteristic	Higher rate facilities, *n*, % or median (IQR) (*N* = 127)	Lower rate facilities, *n*, % or median (QR) (*N* = 499)	*p*
% White residents^a^	46.9 (25.3–68.8)	83.1 (58.9–95.3)	<0.001
% African‐American residents^a^	30.7 (14.3–62.5)	6.4 (1.4–20.0)	<0.001
% male residents^a^	39.6 (32.5–46.2)	30.1 (24.1–38.4)	<0.001
Presence of a resident with a multidrug‐resistant organism	62 (48.8)	176 (35.3)	0.005
Presence of a resident with tracheostomy	50 (39.4)	91 (18.2)	<0.001
% residents with nasogastric or percutaneous gastrostomy tube^a^	7.1 (3.8–11.2)	2.5 (0.7–5.3)	<0.001
% residents with indwelling urinary catheter^a^	6.2 (4.1–8.0)	4.3 (2.6–6.4)	<0.001
% residents with a wound^a^	14.0 (11.0–17.4)	11.8 (7.9–15.3)	<0.001
% residents receiving antibiotics recently^a^	13.2 (8.7–17.5)	11.3 (7.2–15.8)	0.01
% residents on dialysis^a^	2.6 (1.0–4.7)	0.6 (0.0–2.1)	<0.001
% residents with diabetes^a^	39.1 (32.5–43.6)	29.8 (23.4–36.7)	<0.001
% short stay residents (in facility <100 days)^a^	38.3 (30.8–46.1)	30.8 (24.1–39.3)	<0.001
% residents needing extensive assistance for bed repositioning^a^	72.3 (63.8–81.2)	69.8 (58.9–79.7)	0.03
Registered nurse staffing (hours/resident/day)	0.78 (0.61–1.00)	0.79 (0.61–1.05)	0.74
Centers for Medicare & Medicaid Services survey rating^a^	2.5 (1–4)	3 (2–4)	0.001
Centers for Medicare & Medicaid Services quality measure rating^a^	4 (3–5)	4 (3–5)	0.47
Site			
California	28 (22.1)	91 (18.2)	<0.001
Connecticut	21 (16.5)	207 (41.5)	
Georgia	32 (25.2)	37 (7.4)	
Maryland	35 (27.6)	37 (7.4)	
Minnesota	6 (4.7)	80 (16.0)	
New York	4 (3.2)	28 (5.6)	
Tennessee	1 (0.8)	19 (3.8)	

Abbreviation: IQR, interquartile range.

^a^ Median (IQR).

**TABLE 2:** Variation in resident characteristics, nursing homes within the Emerging Infections Program (EIP) surveillance area, 2011–2015.

Facility characteristic	Min	25th percentile	Median	75th percentile	Max
% White residents	0	50	77	93	100
% African‐American residents	0	2	10	28	97
% male residents	0	25	32	41	100
% residents with nasogastric or percutaneous gastrostomy tube	0	1	3	7	93
% residents with indwelling urinary catheter	0	3	5	7	36
% residents with a wound	0	9	12	16	45
% residents receiving antibiotics recently	0	8	12	16	76
% residents on dialysis	0	0	1	3	32
% residents with diabetes	2	25	31	39	93
% short stay residents (in facility <100 days)	0	25	32	41	100
% residents needing extensive assistance for bed repositioning	0	59	70	80	100
Registered nurse staffing (hours/resident/day)	0.17	0.61	0.79	1.04	3.90
Centers for Medicare & Medicaid Services survey rating	1	2	3	4	5
Centers for Medicare & Medicaid Services quality measure rating	1	2	4	5	5

In multivariable regression, a higher percentage of White residents in a facility and a higher number of RN hours per resident per day were associated with lower odds of having a high invasive MRSA rate (Table 3). Presence of a patient with a multidrug‐resistant organism; higher hospital‐onset MRSA rate in the surrounding area; and higher percentages of male residents, residents with a nasogastric or percutaneous gastrostomy tube, short stay residents (i.e., in the facility <100 days), and residents needing extensive assistance for bed repositioning were associated with higher odds of having a high invasive MRSA rate. Variables that were not significantly associated with having higher invasive MRSA rates include percentages of residents that are African American, percentage of residents with diabetes, presence of any resident with tracheostomy, percentage of residents with a wound, percentage of residents receiving antibiotics recently, percentage of residents on dialysis, CMS survey rating, and CMS quality measure rating.

**TABLE 3:** Adjusted odds ratios and 95% confidence intervals for facility characteristics associated with high rates of invasive methicillin‐resistant *Staphylococcus aureus* (MRSA) infections, Emerging Infections Program (EIP) surveillance area, 2011–2015.

Facility characteristic	Adjusted odds ratio (95% confidence interval)	*p*
% White residents^a^	0.82 (0.71–0.95)	0.01
% African‐American residents^a^	1.05 (0.90–1.23)	0.52
% male residents^a^	1.43 (1.13–1.81)	0.003
Presence of a resident with a multidrug‐resistant organism	2.22 (1.36–3.60)	0.001
Presence of a resident with tracheostomy	0.96 (0.47–1.94)	0.91
% residents with nasogastric or percutaneous gastrostomy tube^a^	1.98 (1.05–3.73)	0.03
% residents with a urinary catheter^a^	1.25 (0.55–2.82)	0.59
% residents with a wound^a^	1.26 (0.84–1.90)	0.27
% residents receiving antibiotics recently^a^	0.81 (0.48–1.35)	0.41
% residents on dialysis^a^	3.51 (0.78–15.84)	0.10
% residents with diabetes^a^	1.10 (0.88–1.37)	0.42
% short stay residents (in facility <100 days)^a^	1.68 (1.48–1.91)	<0.0001
% residents needing extensive assistance for bed repositioning^a^	1.26 (1.04–1.52)	0.02
Registered nurse staffing (hours/resident/day)	0.21 (0.09–0.49)	0.0003
Centers for Medicare & Medicaid Services survey rating	0.90 (0.75–1.07)	0.24
Centers for Medicare & Medicaid Services quality measure rating	1.19 (0.92–1.55)	0.18
Hospital‐onset MRSA rate in surveillance area^b^	1.20 (1.10–1.32)	0.0001

^a^ Adjusted odds ratio reflects the effect of increasing the % of residents in a facility with that characteristic by 10%.

^b^ Adjusted odds ratio reflects the effect of increasing the hospital‐onset MRSA rate in the surveillance area by one case per 100,000 population.

## DISCUSSION

Substantial variation in invasive MRSA rates was observed across the nursing homes included in this project. For the most part, facility characteristics associated with high rates reflected increased patient complexity. In addition, lower RN staffing levels were found to be significantly associated with high rates of invasive MRSA infection.

The finding that facilities with patients who have more functional needs had high invasive MRSA rates is consistent with a prior finding that functional independence is protective against MRSA colonization for nursing home residents.^19^ Surprisingly, other than feeding tubes we did not find other medical device use or wounds to be associated with high rates of invasive MRSA, even though they have been associated with MRSA colonization in multiple studies.^19^, ^20^, ^21^, ^22^ Because wounds and other devices were significantly associated with differences in invasive MRSA rates in univariate analysis, this is because of incorporation of other variables in the model (e.g., short stay, limited mobility) that account for the risk associated with devices. Most of the factors associated with high rates were characteristics of the patient population and not directly modifiable by facilities. We also did not find dialysis to be associated with high rates in the model, even though hemodialysis patients are known to be at very high risk for *S. aureus* bloodstream infections.^23^ This might be because only a small proportion of facilities had a substantial proportion of dialysis patients. Indeed, the aOR for the dialysis variable in the model was large but did not reach statistical significance.

However, the finding that increased RN staffing was associated with lower rates of invasive MRSA suggests there may be actions that nursing homes can take to reduce their rates. Less infection control training and increased staff turnover have been associated with infection control citations.^24^, ^25^ CMS has reported that higher staffing levels were associated with improvements in quality measures such as urinary tract infections, sepsis, and electrolyte imbalance.^26^ RN staffing might represent more than just increased availability of hours for direct resident care, given that the actual number of RN staffing hours per resident per day was relatively low. Instead, it may also represent increased availability of staff with a higher level of clinical training who might be able to provide guidance and facility‐wide training on infection control policies or characteristics of facility leadership. For example, increased accessibility of managers and leadership support of staff have been associated with lower staff turnover rates and better resident safety in recent studies,^27^, ^28^ and these benefits might also translate to infection control practices. Of note, the staffing was not significantly different between facilities with low vs high rates in unadjusted analysis, only after adjusting for characteristics of the resident population. This suggests that facilities with more complex resident populations might benefit from higher staffing levels.

The significance—and even lack of significance in some cases—for other factors assessed here might also be important. For example, facilities with a higher proportion of White residents had lower rates. This finding is a potential marker for issues related to health equity that should be explored in future studies. Notably, this finding was independent of resident characteristics and facility staffing levels and parallels recent findings correlating resident racial composition with differences in outcomes.^29^, ^30^ We did not find CMS survey or quality ratings to be associated with differences in invasive MRSA rates. This might be because these ratings do not measure the differences in infection prevention and control quality that are most influential in affecting MRSA rates. Finally, we did find that higher hospital‐onset invasive MRSA rates for the surveillance area were correlated with higher nursing home‐onset invasive MRSA rates. This may reflect the differences in the likelihood that short‐stay nursing home patients, expected to be at the highest risk for invasive nursing home‐onset MRSA, might have acquired MRSA colonization during hospitalization before admission to the nursing home and likely explains some of the regional differences in nursing home‐onset invasive MRSA rates.

This analysis is subject to limitations. The definition of residence in the surveillance area used by this EIP activity excludes cases in persons who moved to a nursing home from outside the surveillance area less than 2 weeks before the MRSA culture, and the impact of this nuance on the results is not known. The facilities within the surveillance area might not be representative of nursing homes nationally. The CMS MDS data are not validated, and the point prevalence data from 2015 used from MDS to characterize facilities might not be reflective of the entire surveillance period. Some data elements that would be useful, such as the presence of central venous catheters or MRSA colonization specifically, were not available in the MDS data and could be unmeasured confounders. The ecological nature of the analysis could limit our ability to precisely understand the individual‐level characteristics of patients. For a small percentage of long‐term care facility‐onset invasive MRSA cases, we were not able to determine the specific facility, so we might have underestimated rates for some facilities. The data from this time period might not reflect more recent trends. Finally, this article uses a relatively simple logistic regression model to describe the factors associated with differences in rates of invasive MRSA infections. In the future, for a specific application that needs to identify facilities most likely to have high rates, another type of model using more sophisticated machine learning approaches might yield even more accurate predictions.^31^

In conclusion, in this analysis of invasive MRSA infection data from >600 nursing homes, we found several factors to be associated with high rates of nursing home‐onset invasive MRSA infection, including medical complexity of residents and lower RN staffing hours per resident. These findings suggest potential opportunities to improve infection prevention practices in US nursing homes in general, such as increased focus on residents needing the most assistance with activities of daily living. In addition, the model used in this analysis could help to distinguish nursing homes likely to have high rates from those likely to have lower rates; this could be particularly useful for public health and healthcare organizations seeking to allocate limited resources and prioritize specific nursing homes with the highest burden of invasive MRSA infections for future prevention efforts.

## AUTHOR CONTRIBUTIONS

All authors made substantial contributions to conception and design, or acquisition of data, or analysis and interpretation of data, drafting the article or revising it critically for important intellectual content, and final approval of the version to be published. Conception and drafting: Nimalie Stone, Isaac See, and Kara Slifka Jacobs. Data acquisition: Carmen Bernu, Ghinwa Dumyati, Anita Gellert, Lee H. Harrison, Laura Jeffrey, Ruth Lynfield, Tiffanie Markus, Joelle Nadle, Susan Petit, Susan Ray, William Schaffner, Isaac See, and Prabasaj Paul. Analysis: Isaac See, Rongxia Li, Kelly M. Hatfield, Runa Gokhale, Kelly A. Jackson, and Prabasaj Paul. Interpretation and revising: All authors.

## CONFLICT OF INTEREST STATEMENT

Nothing to disclose.

## SPONSOR'S ROLE

The CDC oversaw all aspects of the study other than subject recruitment and directly determined the design, methods, analysis, and preparation.

## FINANCIAL DISCLOSURE

This work was supported by a cooperative agreement through the CDC EIP (Grants U50CK000201 [California], U50CK000195 [Connecticut], U50CK000196 [Georgia], U50CK000203 [Maryland], U50CK000204 [Minnesota], U50CK000199 [New York], and U50CK000198 [Tennessee]).

## Supporting information


**Supplemental Table 1.** Facility variables obtained from the Centers for Medicare & Medicaid Services (CMS) Minimum Data Set and CMS nursing home compare and the conceptual meaning of the variable as related to invasive methicillin‐resistant *Staphylococcus aureus* (MRSA) rates.
**Supplemental Figure 1.** Scatter plot of invasive community‐associated (CA) and hospital‐onset (HO) methicillin‐resistant *Staphylococcus aureus* rates for the 7 Emerging Infections Program Sites, 2014.
**Supplemental Figure 2.** Distribution of nursing home‐onset invasive methicillin‐resistant *Staphylococcus aureus* (MRSA) cases, nursing homes in the Emerging Infection Program surveillance area, 2011–2015.

## Endnote

^1^
See e.g., 45 C.F.R. part 46.102(l) (2), 21 C.F.R. part 56; 42 U.S.C. §241(d); 5 U.S.C. §552a; 44 U.S.C. §3501 et seq.
