# Supplementary material for: Characteristics of nursing homes with high rates of invasive methicillin‐resistant Staphylococcus aureus infections
Source: J Am Geriatr Soc. 2025 Jan 20;73(3):849–58. doi: 10.1111/jgs.19189 (PMC11907741; doi:10.1111/jgs.19189)

Appendix

Supplemental methods

Surveillance area

The counties that invasive MRSA surveillance was conducted in for this project were as follows:

California: Alameda, Contra Costa, San Francisco

Connecticut: all (entire state)

Georgia: Clayton, Cobb, Dekalb, Douglas, Fulton, Gwinnett, Newton, Rockdale

Maryland: Baltimore County, Baltimore City

Minnesota: Hennepin, Ramsey

New York: Monroe

Tennessee: Davidson

Epidemiologic definitions

Hospital-onset (HO) cases were defined as having the initial invasive MRSA culture obtained on the fourth day or later of a hospitalization. Community-associated (CA) cases were defined as not being HO **and** the patient not having any of the following: hospitalization, nursing home residence, surgery, or dialysis in the year prior to specimen collection; or a central venous catheter within the 2 days prior to specimen collection.

Data sources and identification of facilities

Nursing homes within the surveillance area were identified from the Centers for Medicare & Medicaid Services (CMS) Provider of services (POS) Hospital & non-Hospital facilities file. The POS file is updated quarterly and for each year the fourth quarter update was used to identify nursing homes open that year. Nursing homes were selected based on having a “provider category code” of 02 (Skilled Nursing Facility/Nursing Facility [Dually Certified]), 03 (Skilled Nursing Facility/Nursing Facility [Distinct Part]), 04 (Skilled Nursing Facility), or 10 (Nursing Facility), and were determined to be within the surveillance area based on state and FIPS county codes.

In addition, the POS file was reviewed to find counties with nursing homes located in different cities or zip codes. In these instances, the facility data were manually reviewed to verify the correct county information. The same process was conducted for the CMS cost reports.

When facilities closed and re-opened with a different CMS certification number (CCN) at the same location, then case counts and resident-day denominators from the two locations were merged together.

Selection of facility characteristics

A full list of facility-level variables obtained from the Minimum Data Set (MDS) or nursing home compare are listed in Table S1 along with how the potential relationship to invasive methicillin-resistant *Staphylococcus aureus* (MRSA) rates was considered conceptually. When more than one variable represented the same concept and was considered to be directly related to other variables in that concept category, the variable with the most clear interpretability was chosen. For example, the proportion of patients in the facility with varying length of stay was calculated in several ways (e.g., % <30 days, % 30-49 days) to potentially represent the percent that were short stay residents, and for the concept of short stay, the proportion of patients in the facility <100 days was chosen because that aligned with CMS definitions of short stay.

Imputation of missing resident-day data

Resident-day data that were missing from the CMS cost reports were imputed based on the distribution of known resident-days and the bed size from both the cost reports and the POS file. Imputation was conducted using the SAS proc MI procedure and the predictive mean matching method. Five imputations were conducted and the mean of the imputed resident-days was used for analysis.

Supplemental Table 1: Facility variables obtained from the Centers for Medicare & Medicaid Services (CMS) Minimum Data Set and CMS nursing home compare and the conceptual meaning of the variable as related to invasive methicillin-resistant *Staphylococcus aureus* (MRSA) rates

**Description of variable**	**Variable conceptual meaning**
% male	Patient demographics
% American Indian	Patient demographics
% Asian	Patient demographics
% African-American	Patient demographics
% Hispanic	Patient demographics
% Native Alaskan/Hawaiian	Patient demographics
% White	Patient demographics
% admission (vs reentry)	Short stay concept
% with indwelling urinary catheter	Device use (colonization)
% with external urinary catheter	Device use (colonization)
% with ostomy	Device use (colonization)
% with intermittent catheterization	Device use (colonization)
% with any type of urinary appliance or ostomy	Device use (colonization)
% with heart failure	Comorbidity risk for infection
% with multidrug-resistant organism (MDRO) in past 7 days	MDRO (transmission)
% with pneumonia in past 7 days	antibiotic use surrogate (colonization)
% with septicemia diagnosed in past 7 days	antibiotic use surrogate (colonization)
% with urinary tract infection diagnosed in past 30 days	antibiotic use surrogate (colonization)
% with viral hepatitis in past 7 days	Unclear
% with wound infection (other than foot) in past 7 days	wound (colonization, transmission)
% with diabetes	Comorbidity risk for infection
% with hip fracture relating to current status	Functional status (transmission risk)
% with other fractures	Functional status (transmission risk)
% with Alzheimer's disease	Short stay concept
% with cerebral palsy	Functional status (transmission risk)
% with stroke	Short stay concept
% with non-Alzheimer's dementia	Short stay concept
% with hemiplegia or hemiparesis	Functional status (transmission risk)
% with paraplegia	Functional status (transmission risk)
% with quadriplegia	Functional status (transmission risk)
% with multiple sclerosis	Functional status (transmission risk)
% with Parkinson's disease	Short stay concept
% with seizure disorder or epilepsy	Unclear
% with traumatic brain injury	Short stay concept
% with asthma	Unclear
% with respiratory failure in past 7 days	antibiotic use surrogate (colonization)

% with less than 6 months life expectancy	Comorbidity risk for infection
% losing liquids or solids from mouth when eating or drinking	Unclear
% holding food in mouth/cheeks or residual food in mouth after meals	Unclear
% coughing or choking during meals or when swallowing medications	Unclear
% complaining of difficulty or pain with swallowing	Unclear
% with any swallowing disorder	Unclear
% receiving parenteral feeding in past 7 days	Device use (infection)
% with nasogastric or percutaneous gastrostomy (PEG) tube in past 7 days	Device use (colonization)
% with any nutritional approach (parenteral feeding, feeding tube, mechanically altered diet, therapeutic diet) in past 7 days	Unclear
% at risk of developing pressure ulcers	wound (colonization, transmission)
% with stage 1 or higher pressure ulcer	wound (colonization, transmission)
% with foot infection	wound (colonization, transmission)
% with diabetic foot ulcer	wound (colonization, transmission)
% with other open lesion of the foot	wound (colonization, transmission)
% with open lesion other than ulcers, rashes, cuts	wound (colonization, transmission)
% with surgical wound	wound (colonization, transmission)
% with burn (second or third degree)	wound (colonization, transmission)
% with skin tear	wound (colonization, transmission)
% with moisture-associated skin damage	wound (colonization, transmission)
% with any skin problem (other than decubitus ulcer, venous/arterial ulcers)	wound (colonization, transmission)
% receiving chemotherapy in last 14 days while a resident	Device use (infection) and comorbidity risk for infection
% receiving tracheostomy care in last 14 days while a resident	Device use (colonization)
% receiving ventilator or respiratory care in last 14 days while a resident	Device use (colonization)
% receiving intravenous medication in last 14 days while a resident	Device use (infection)
% receiving dialysis in last 14 days while a resident	Device use (infection)
% on isolation quarantine for active infectious diseases in last 14 days while a resident	MDRO (transmission)
% receiving flu vaccine in facility (excluding those who received vaccine outside or had contraindication)	Facility quality of care
% receiving pneumococcal vaccine (out of those eligible)	Facility quality of care
% ≥65 years old	Short stay concept
% entered from community (including assisted living)	Short stay concept
% entered from another nursing home or swing bed	Short stay concept
% entered from acute care hospital	Short stay concept

% entered from psychiatric hospital	Short stay concept
% entered from inpatient rehabilitation facility	Short stay concept
% entered from Intellectual Disability (ID)/Developmntal Disability (DD) facility	Short stay concept
% entered from hospice	Short stay concept
% entered from long-term care hospital	Short stay concept
% entered from other place	Short stay concept
% within 30 days of hospitalization	Short stay concept
% in the facility < 30 days	Short stay concept
% in the facility 30-49 days	Short stay concept
% in the facility 50-99 days	Short stay concept
% in the facility 100-179 days	Short stay concept
% in the facility 180-364 days	Short stay concept
% in the facility 365+ days	Short stay concept
% in the facility <100 days	Short stay concept
% with hemiplegia/hemiparesis, paraplegia, or quadriplegia	Functional status (transmission risk)
% with any chewing or swallowing disorder	Unclear
% admission assessment	Short stay concept
% quarterly assessment	Short stay concept
% annual assessment	Short stay concept
% change in status assessment	Short stay concept
% correction to prior comprehensive assessment	Short stay concept
% correction to prior quarterly assessment	Short stay concept
% no Omnibus Budget Reconciliation Act (OBRA) reason for assessment	Short stay concept
% needing extensive assistance or total dependence for bed mobility	Functional status (transmission risk)
% needing extensive assistance or total dependence for transfers	Functional status (transmission risk)
% needing extensive assistance or total dependence to walk in room	Functional status (transmission risk)
% needing extensive assistance or total dependence to walk in corridor	Functional status (transmission risk)
% needing extensive assistance or total dependence for locomotion on unit	Functional status (transmission risk)
% needing extensive assistance or total dependence for locomotion off unit	Functional status (transmission risk)
% needing extensive assistance or total dependence for dressing	Functional status (transmission risk)
% needing extensive assistance or total dependence on eating	Functional status (transmission risk)
% needing extensive assistance or total dependence on toilet use	Functional status (transmission risk)

% needing extensive assistance or total dependence for personal hygiene	Functional status (transmission risk)
% with score of 3 or higher on activities of daily living (ADL) hierarchy scale	Functional status (transmission risk)
% with a stage 2 pressure ulcer	wound (colonization, transmission)
% with a stage 3 pressure ulcer	wound (colonization, transmission)
% with a stage 4 pressure ulcer	wound (colonization, transmission)
% with a stage 2 or higher pressure ulcer	wound (colonization, transmission)
% with an arterial ulcer	wound (colonization, transmission)
% with any wound (excluding burn or surgical wound)	wound (colonization, transmission)
% receiving an antibiotic in past 7 days	antibiotic use surrogate (colonization)
average length of stay (for residents in 2015)	Short stay concept
Reported Nurse Aide Staffing - Hours per Resident per Day	Facility staffing
Reported licensed professional nurse (LPN) Staffing - Hours per Resident per Day	Facility staffing
Reported registered nurse (RN) Staffing - Hours per Resident per Day	Facility staffing
Reported Licensed Staffing - Hours per Resident per Day (RN + LPN)	Facility staffing
Reported Total Nurse Staffing - Hours per Resident per Day (Aide+LPN+RN)	Facility staffing
Reported Physical Therapy Staffing - Hours per Resident Per Day	Facility staffing
Expected nurse aid staffing - hours per resident per day	Facility staffing
Expected LPN staffing - hours per resident per day	Facility staffing
Expected RN staffing - hours per resident per day	Facility staffing
Expected Total nurse staffing - hours per resident per day (RN + LPN)	Facility staffing
Adjusted Nurse Aide Staffing - Hours per Resident per Day	Facility staffing
Adjusted LPN Staffing - Hours per Resident per Day	Facility staffing
Adjusted RN Staffing - Hours per Resident per Day	Facility staffing
Adjusted Total Nurse Staffing - Hours per Resident per Day (Aide+LPN+RN)	Facility staffing
Overall CMS star rating	Facility quality of care
CMS survey rating	Facility quality of care
CMS quality metric rating	Facility quality of care
CMS staffing rating	Facility staffing
CMS RN staffing rating	Facility staffing

Supplemental Figure 1: Scatter plot of invasive community-associated (CA) and hospital-onset (HO) methicillin-resistant *Staphylococcus aureus* rates for the 7 Emerging Infections Program Sites, 2014**.**

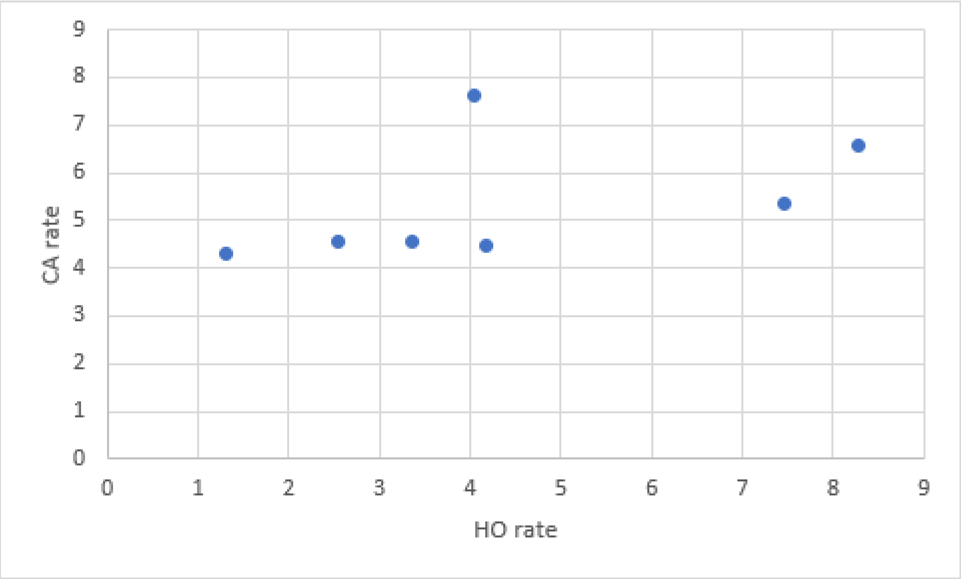

Supplemental Figure 2: Distribution of nursing home-onset invasive methicillin-resistant *Staphylococcus aureus* (MRSA) cases, nursing homes in the Emerging Infection Program surveillance area, 2011-2015

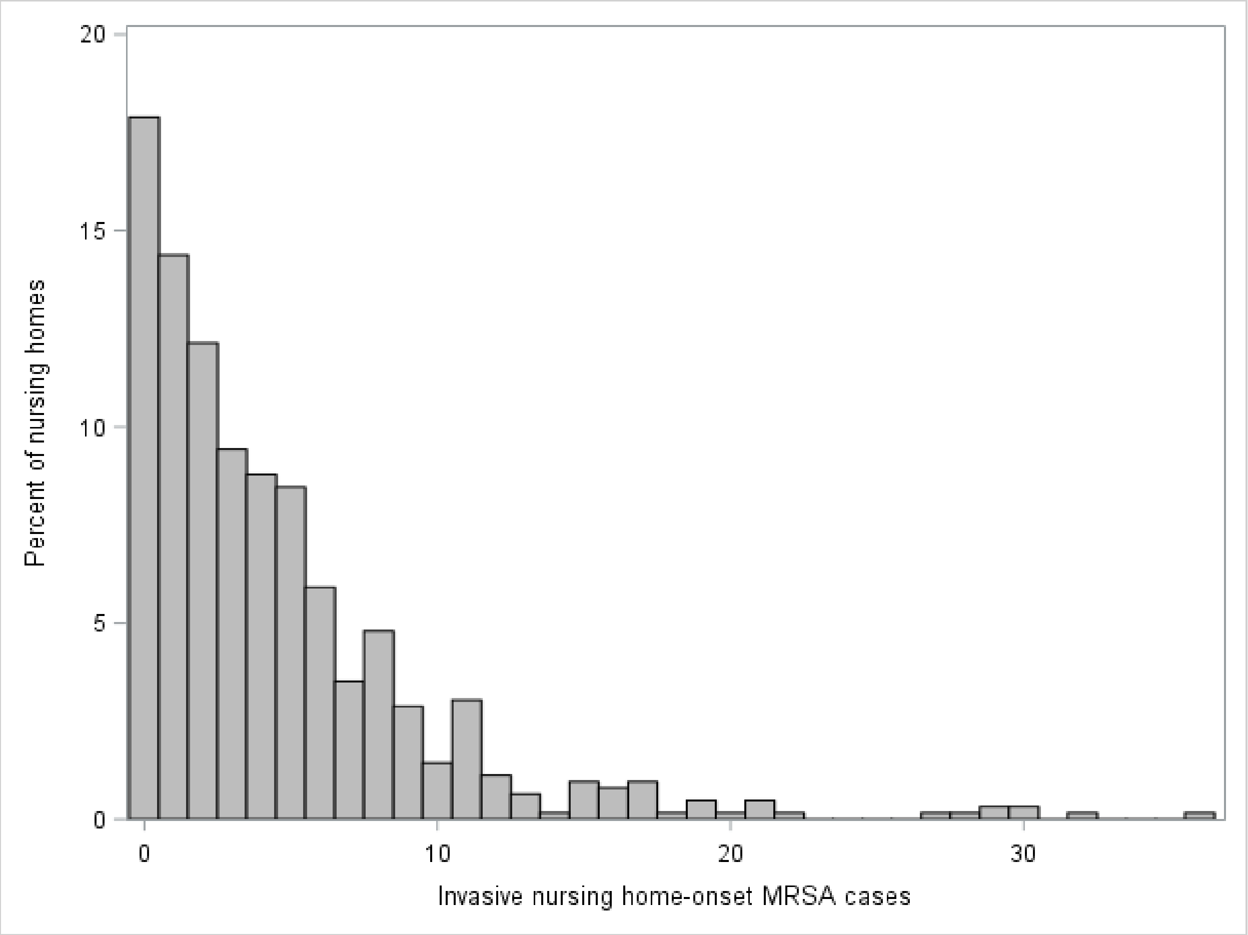

Supplement: Supplementary file 1 — Supplemental Table 1. Facility variables obtained from the Centers for Medicare & Medicaid Services (CMS) Minimum Data Set and CMS nursing home compare and the conceptual meaning of the variable as related to invasive methicillin‐resistant Staphylococcus aureus (MRSA) rates. Supplemental Figure 1. Scatter plot of invasive community‐associated (CA) and hospital‐onset (HO) methicillin‐resistant Staphylococcus aureus rates for the 7 Emerging Infections Program Sites, 2014. Supplemental Figure 2. Distribution of nursing home‐onset invasive methicillin‐resistant Staphylococcus aureus (MRSA) cases, nursing homes in the Emerging Infection Program surveillance area, 2011–2015. [file JGS-73-849-s001.pdf]
